# A phase I dose-escalation study of PEP02 (irinotecan liposome injection) in combination with 5-fluorouracil and leucovorin in advanced solid tumors

**DOI:** 10.1186/s12885-016-2933-6

**Published:** 2016-11-21

**Authors:** Nai-Jung Chiang, Tsu-Yi Chao, Ruey-Kuen Hsieh, Cheng-Hsu Wang, Yi-Wen Wang, C. Grace Yeh, Li-Tzong Chen

**Affiliations:** 1 National Institute of Cancer Research, National Health Research Institutes, 2F, No. 367, Sheng-Li Road, Tainan, 704 Taiwan; 2 Division of Hematology/Oncology, Department of Internal Medicine, National Cheng Kung University Hospital, Tainan, Taiwan; 3 Division of Hematology and Oncology, Taipei Medical University-Shuang Ho Hospital, Taipei, Taiwan; 4 Division of Hematology and Oncology, Department of Internal Medicine, Mackay Memorial Hospital, Taipei, Taiwan; 5 Division of Hematology/Oncology, Department of Internal Medicine, Chang Gung Memorial Hospital, Linkou, Taiwan; 6 PharmaEngine, Inc, Taipei, Taiwan; 7 Department of Internal Medicine, Kaohsiung Medical University Hospital, Kaohsiung Medical University, Kaohsiung, Taiwan

**Keywords:** Liposomal irinotecan, 5-fluorouracil, Dose-limiting toxicity, Maximum tolerated dose

## Abstract

**Background:**

PEP02 (also known as MM-398, nal-IRI) is a novel nanoparticle formulation of irinotecan encapsulated in liposomes. The aims of this study were to investigate the dose-limiting toxicity (DLT), maximum tolerated dose (MTD) and pharmacokinetics (PK) of PEP02 in combination with 5-FU and LV, in patients with advanced refractory solid tumors.

**Methods:**

Patients were enrolled in cohorts to receive PEP02 from 60 to 120 mg/m^2^ (dose expressed as the irinotecan hydrochloride trihydrate salt) as a 90-min intravenous infusion on day 1, followed by 24 h infusion of 5-FU 2,000 mg/m^2^ and LV 200 mg/m^2^ on days 1 and 8, every 3 weeks.

**Results:**

A total of 16 patients were assigned to four dose levels, 60 (three patients), 80 (six patients), 100 (five patients) and 120 mg/m^2^ (two patients). DLT was observed in four patients, two at the 100 mg/m^2^ dose level (one had grade III infection with hypotension and grade III hemorrhage; the other had grade III diarrhea and grade IV neutropenia), and two at the 120 mg/m^2^ dose level (one had grade III diarrhea and grade IV neutropenia; the other had grade III diarrhea). The MTD of PEP02 was determined as 80 mg/m^2^. The most common treatment-related adverse events were nausea (81%), diarrhea (75%) and vomiting (69%). Among the six patients who received the MTD, one patient exhibited partial response, four patients had stable disease and one showed progressive disease. Pharmacokinetic data showed that PEP02 had a lower peak plasma concentration, longer half-life, and increased area under the plasma concentration-time curve from zero to time t of SN-38 than irinotecan at similar dose level.

**Conclusions:**

The MTD of PEP02 on day 1 in combination with 24-h infusion of 5-FU and LV on days 1 and 8, every 3 weeks was 80 mg/m^2^, which will be the recommended dose for future studies.

**Trial registration:**

The trial was retrospectively registered (NCT02884128) with date of registration: August 12, 2016.

**Electronic supplementary material:**

The online version of this article (doi:10.1186/s12885-016-2933-6) contains supplementary material, which is available to authorized users.

## Background

PEP02 (also known as MM-398, nal-IRI) is an encapsulated nanoliposomal formulation of irinotecan hydrochloride (CPT-11) [1]. Irinotecan is a water-soluble semi-synthetic analogue of the natural alkaloid, camptothecin. It prevents DNA from unwinding and replication by inhibition of topoisomerase-I, and has already been approved for use worldwide. However, at higher dosage, irinotecan causes severe diarrhea and myelosuppression, which limits its therapeutic index. The therapeutic benefits of encapsulating anti-cancer drugs such as daunorubicin, doxorubicin and cytarabine in liposomes have been documented [2]. An appropriately designed liposome formulation may reduce the toxicity of cytotoxic agents to healthy tissues while maintaining its anti-tumor potency, which in turn improves treatment efficacy.

In our previous study, the maximum tolerated dose (MTD) of PEP02 monotherapy was found to be 120 mg/m^2^ at 3-week interval with favorable pharmacokinetic (PK) parameters of the active metabolite, SN-38 [3]. The acceptable toxicity profile explains the beneficial effects of PEP02 in combination with other cytotoxic agents. Irinotecan in combination with 5-fluorouracil (5-FU) and leucovorin (LV) is the first-line or second-line therapy for locally advanced and metastatic colorectal cancer [4]. A synergistic effect was observed upon the sequential administration of irinotecan and 5-FU [5, 6]. On the basis of these results, the combination of PEP02 with 5-FU and LV is considered a reasonable approach to enhance their therapeutic efficacy. This Phase I dose escalation study aimed to investigate the MTD, dose-limiting toxicity (DLT) and recommended dose of PEP02 in combination with 5-FU and LV.

Irinotecan is converted by carboxylesterases to its potent metabolite, SN-38, which is detoxified in part by converting to inactive SN-38 glucuronide (SN-38G) through UDP-glucuronosyl transferase 1A isoforms (UGT1A) [7]. The activity of UGT1A is related to gene polymorphism of *UGT1A* family members. Individuals with genetic mutations of *UGT1A* exhibit reduced glucuronidation of SN-38 and an elevated risk of neutropenia and diarrhea compared with patients with wild-type alleles [8]. The correlation of *UGT1A* polymorphisms and toxicities is discussed.

## Methods

### Patient eligibility

This trial was a multi-center, open-label, Phase I, dose escalation study of PEP02 (PharmaEngine, Inc., Taipei, Taiwan) in combination with 5-FU and LV in patients with advanced solid tumors. The inclusion criteria were as follows: (1) histologically or cytologically confirmed advanced solid tumor refractory to standard systemic chemotherapy; (2) aged between 20 and 70 years; (3) Eastern Cooperative Oncology Group performance score (ECOG PS) of 0 or 1; (4) life expectancy ≥ 2 months; (5) adequate bone marrow, hepatic and renal functions: white blood cells ≥ 3,000/mm^3^, absolute neutrophil count ≥ 1,500/mm^3^, platelets ≥ 100,000/mm^3^, hemoglobin ≥ 10 g/dL, serum total bilirubin within normal range, AST and ALT ≤ 3× upper limit of normal range, serum creatinine ≤ 1.5 mg/dL and blood urea nitrogen ≤ 25 mg/dL; (6) no prior treatment for at least 4 weeks before study initiation, including major surgery, chemotherapy, any investigational products or radiotherapy (6 weeks for nitrosoureas and mitomycin C); (7) recovered from all treatment-related toxicities or resolved to no greater than grade 1 before enrollment; and (8) written informed consent.

The exclusion criteria were as follows: (1) known or suspicious primary or secondary brain tumors; (2) HBsAg-positive or anti-HCV antibody-positive with splenomegaly (defined as spleen size > 11 cm measured in longest diameter by CT scan); (3) uncontrolled active infection or other concomitant serious disease; (4) pregnancy or breast-feeding; (5) previous exposure to irinotecan; (6) history of allergic reactions to compounds of similar chemical or biologic composition as PEP02, 5-FU, or LV. This trial was approved by the independent ethics committee of each participating institute and the Department of Health, Executive Yuan, Taiwan, and was performed in accordance with International Conference on Harmonization Good Clinical Practice guidelines and Good Clinical Laboratory Practice.

### Treatment and dose escalation schedule

The study had a traditional 3 + 3 design with three-patient cohorts for each dose level. Dose escalation was only performed after the successful completion of at least 1 full 3-week cycle by each patient in the dosing cohort. If none of the first three patients experienced DLT, dose escalation was carried out for the next cohort of patients. If one of three patients developed DLT, the cohort was expanded to six patients. If two or more patients experienced any DLT, no more patients were to be entered at the current dose level and the lower dose level was to be declared the MTD. The MTD was the highest dose level with no more than 1 DLT among the accruals. A minimum of six patients were required to be tested at the dose level defined as the MTD. The starting dose of PEP02 was 60 mg/m^2^ with dose expressing as the irinotecan hydrochloride trihydrate salt, which was escalated by increments of 20 mg/m^2^ between dose levels. Each patient was assigned to a dose level, and no intra-patient dose escalation was allowed. 5-FU and LV were administered at a fixed dose of 2000 and 200 mg/m^2^, respectively. PEP02 was administered by intravenous infusion over 90 min on Day 1, followed by 24-h intravenous infusion of 5-FU and LV on days 1 and 8 every 3 weeks. Pre-medication included dexamethasone and a serotonin-antagonist. Prophylactic anti-cholinergic agent was not administered unless acute cholinergic reaction was observed in prior cycles of treatment. Anti-diarrhea agents were started according to the guideline of American Society of Clinical Oncology. Treatment was continued to a maximum of 6 cycles or until disease progression, unacceptable toxicity, treatment delay > 2 weeks, or patient’s refusal or death.

Dose modification on day 1 of subsequent cycles was only applied to PEP02, while the dosage of 5-FU/LV remained unchanged. All dose modifications were to be based on the worst proceeding toxicity. For patients who experienced ≥ grade 3 hematologic or non-hematologic toxicities, the dose of PEP02 was reduced by one dose level. In addition, the dose of 5-FU on day 8 of each cycle could be adjusted according to the laboratory data before the dosing. If the absolute neutrophil count (ANC) is between 1,000 and 1,499/μL, platelet count is between 50,000 and 99,999/μL, or diarrhea of grade 2 severity is observed, the dose of 5-FU could be decreased by 25%. 5-FU was withheld when ANC < 999/μL, platelet count < 50,000/μL or grade 3 diarrhea was observed. The conditions for the administration of the next cycle of treatment were ANC ≥ 1,500/μL, platelet counts ≥ 100,000/μL, serum creatinine ≤ 1.5 mg/dL, and full resolution of gastrointestinal toxicities.

### Definition of dose-limiting toxicity (DLT)

Toxicities were assessed according to the National Cancer Institute’s CTCAE version 3.0 (CTCAE, v3). DLT was defined as occurrence of 1 or more of the following events attributable to the study drugs during the first cycle: (1) grade III or IV non-hematological toxicity, except grade III nausea, vomiting, or anorexia; (2) grade IV hematologic toxicity lasting for ≥3 days; (3) grade III hematologic toxicity associated with complications (e.g. neutropenic fever or bleeding); (4) dose delay of more than 2 weeks owing to drug-related toxicity. In addition, hematological assessment was performed daily whenever grade IV hematological toxicity occurred.

### Patient evaluation

Pretreatment evaluations included medical history, physical examination, performance status, complete blood count, hepatic and renal functions and serology of HBsAg and anti-HCV antibody. Patients were evaluated weekly with complete blood count and biochemistry analysis. Radiologic studies to assess response were performed at baseline and then every 2 cycles of therapy according to the guidelines of Responses Evaluation Criteria in Solid Tumors criteria version 1.0. All complete and partial responses required confirmation by two consecutive observations at least 4 weeks apart.

### Pharmacokinetic sampling and analyzing

During the first cycle of treatment, blood samples were collected before treatment, during the infusion at 30 and 60 min, at the end of infusion, at1, 3, 9, 24, 48, 72 and 168 h after the end of infusion, and before the second cycle. Plasma levels of irinotecan and SN-38 were measured by validated LC/MS/MS analytical methods. The peak plasma concentration (C_max_), time at which C_max_ occurred (T_max_), elimination half-life (t_1/2_), area under the plasma concentration-time curve from zero to time t (AUC_0→t_), AUC through infinite time (AUC_0→∞_), and clearance (CL) were calculated. Pharmacokinetic parameters of individual data set were analyzed by a non-compartmental model by using WinNonlin™ (Centara, St. Louis, MO).

### Pharmacogenetic studies

Additional 5 mL blood sample was collected into a PAXgene vacutainer tube and DNA was extracted using a DNA purification kit. Fragment analysis was used for the detection of short tandem repeat polymorphism. The TaqMan-Allelic discrimination method or direct sequencing was used for the detection of single nucleotide polymorphisms, including UGT1A1*28 and UGT1A1*6.

### Statistical analysis

The statistical analysis was descriptive and any inferential statistics was exploratory in nature. Summary statistics were provided for all efficacy, pharmacokinetic, pharmacogenetic, safety and baseline/demographic variables. For categorical variables, frequency tables including percentages were presented. For continuous variables, descriptive statistics such as number of available observations, mean with standard deviation (STD), minimum, and maximum were tabulated.

## Results

### Patient characteristics, dose escalation, DLT and MTD

Between March 2006 and August 2008, a total of 16 patients (seven men and nine women) were enrolled. The demographics and baseline characteristics of all patients are summarized in Table 1. The median age was 49 years (range: 30–67 years). The most common primary tumors were pancreatic, stomach, and breast carcinomas. Other tumor types included keratinizing squamous cell carcinoma, cervical cancer and nasopharyngeal carcinoma. A total of 66 cycles of treatment were initiated, with an average of 4.1 cycles per patient (range: 1–6 cycles). There were seven patients (43.8%) completed all 6 cycles of treatment.

**Table 1:** Patient characteristics

Characteristic	Patients, n (%)
Patients enrolled	16
Age (yrs)	
Median	49
Range	30–67
Sex	
Male	7 (44)
Female	9 (56)
ECOG performance status	
0	3 (19)
1	13 (81)
Tumor type	
Breast cancer	4 (25)
Pancreatic cancer	5 (31)
gastric cancer	4 (25)
Other	3 (19)
Previous treatment	
Surgery	14 (88)
Radiotherapy	9 (56)
Chemotherapy	16 (100)

*Abbreviation*: *ECOG* Eastern Cooperative Oncology Group

The dose escalation schedule is outlined in Table 2. These patients were assigned to four dose levels, with three, six, five and two patients in dose level I, II, III, and IV, respectively. At first, none of the first three patients experienced DLT at dose level I, II, and III; therefore, the dose level was further escalated to 120 mg/m^2^. Because both of the initial two patients at 120 mg/m^2^ level experienced DLT during the first cycle of treatment (one had grade III diarrhea and grade IV neutropenia; the other had grade III diarrhea), three additional patients were recruited at the prior dose level, 100 mg/m^2^. However, both of the two newly accrued patients at 100 mg/m^2^ level experienced DLTs (one had grade III infection with hypotension and grade III hemorrhage; the other had grade III diarrhea and grade IV neutropenia), resulting in 2 episodes of DLT among the five patients at this dose level. Therefore, the tested dose level was further de-escalated to 80 mg/m^2^. Since none of the patients experienced any DLT, 80 mg/m^2^ of PEP02 by 90-min intravenous infusion was determined as the MTD in combination with weekly infusion of 5-FU/LV on days 1 and 8 of a 21-day cycle.

**Table 2:** Dose escalation scheme

Dose Level	PEP02 (mg/m^2^)	No. patients	No. patients with DLT
I	60	3	0
II	80	3 + 3	0 + 0
III	100	3 + 2	0 + 2
IV	120	2	2

*Abbreviation*: *DLT* dose-limiting toxicity

### Toxicity

All 16 patients were assessed for toxicity. Table 3 summarizes the therapy-induced toxicity during treatment. There were three (18.4%) patients had grade III or above adverse events (AEs), and 13 and 0.2% of AEs led to dosing delay/reduction and permanent discontinuation of treatment, respectively. No treatment-related death was reported in the study.

**Table 3:** Treatment-emergent AEs with maximum CTC grade by dose level (incidence ≥ 20%)

	Total (*N* = 16)	60 mg/m^2^ *N* = 3	80 mg/m^2^ *N* = 6	100 mg/m^2^ *N* = 5	120 mg/m^2^ *N* = 2
AE	All grade	Grade 3–4			
Anemia	7 (43.8%)	0	0	2 (40%)	0
Leukopenia	6 (37.5%)	0	0	2 (40%)	1 (50%)
Neutropenia	6 (37.5%)	1 (33.3%)	1 (16.7%)	2 (40%)	1 (50%)
Abdominal pain	7 (43.8%)	0	0	1 (20%)	1 (50%)
Diarrhea	12 (75.0%)	0	1 (16.7%)	2 (40%)	2 (100%)
Nausea	13 (81.3%)	0	1 (16.7%)	0	0
Vomiting	12 (75.0%)	0	1 (16.7%)	0	0
Fatigue	8 (50.0%)	0	0	1 (20%)	0
Infection	6 (37.5%)	0	0	2 (40%)	1 (50%)
Anorexia	4 (25.0%)	0	0	1 (20%)	0
Hypoalbuminemia	4 (25.0%)	0	1 (16.7%)	0	0
Hypokalemia	8 (50.0%)	1 (33.3%)	2 (33.3%)	2 (40%)	1 (50%)
Hyponatremia	4 (25.0%)	0	0	1 (20%)	1 (50%)
Cough	5 (31.3%)	1 (33.3%)	0	0	0

*Abbreviation*: *AE* adverse event

The most common treatment-related AEs included nausea (81.3% in incidence), followed by diarrhea (75.0%), vomiting (68.8%), fatigue (43.8%), mucositis (mucosa inflammation, 43.8%), leucopenia (37.5%), neutropenia (37.5%), weight loss (37.5%), anemia (31.3%), and alopecia (31.3%). Acute cholinergic reaction was rarely observed. Compared with the entire safety population, patients who received 80 mg/m^2^, the MTD dose of PEP02 experienced less treatment-related AEs (51.1% versus 57.6%), as well as grade III or above AEs (10.6% versus 18.4%).

### Pharmacokinetics and exploratory pharmacogenetic studies

**Fig. 1:**
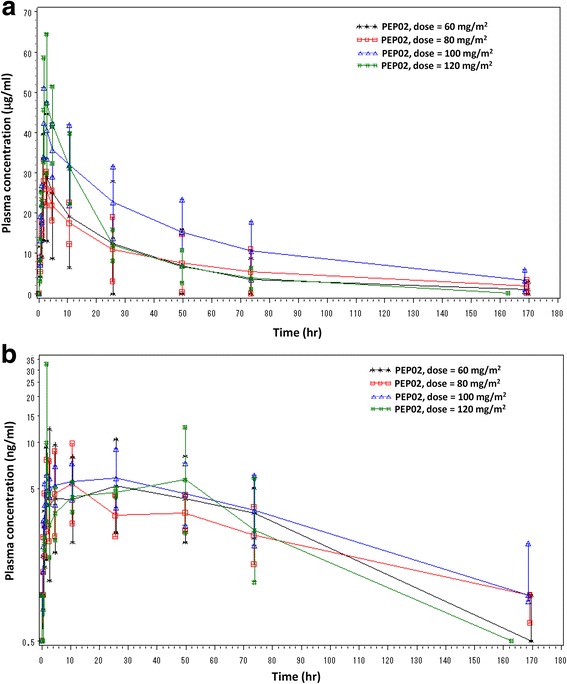
Plasma concentration-time profiles of **a** CPT-11 and **b** SN-38 at different PEP02 doses

The PK of PEP02 is shown in Table 4, Fig. 1a and b. CPT-11 and SN-38 were characterized for PEP02 single dose PK at dose levels of 60, 80, 100, and 120 mg/m^2^ by 90-min intravenous infusion. Changes in the plasma concentration of CPT-11 showed almost the same pattern at all levels. All concentration curves of plasma CPT-11 peaked quickly and reached the maximum around 1 h after the end of PEP02 infusion and gradually dropped in a mono-exponential pattern until the last sampling point, which was similar to that observed for PEP02 monotherapy in a previous study [3]. At the MTD of PEP02, the Cmax of SN-38 was lower (7.98 ± 4.39 ng/ml) than that of the conventional formulation of irinotecan at 125 mg/m^2^ (26.3 ± 11.9 ng/ml), whereas the AUC of SN-38 was higher than that of irinotecan (AUC0 → t: 343.36 ± 133.24 ng/ml*h vs. 229 ± 108 ng/mL*h). The t_1/2_ of SN-38 at the MTD of PEP02 was 57.54 ± 17.81 h, which was relatively longer than that of the conventional formulation (10.4 ± 3.1 h). No statistically significant difference was observed in the mean values of all pharmacokinetic parameters of SN-38 among the 4 dose levels.

**Table 4:** Pharmacokinetic parameters of PEP02 at each dose level

	Dose of PEP02 (mg/m^2^)	*C*_*max*_ CPT-11 (μg/mL) SN-38 (ng/mL)	*T*_*max*_ (hr)	AUC_0→169.5_ CPT-11 (hr-μg/mL) SN-38 (hr-ng/mL)	AUC_0→∞_ CPT-11 (hr-μg/mL) SN-38 (hr-ng/mL)	*V*_*ss*_ (L/m^2^)	Cl (mL/hr/m^2^)	*t*_*1/2*_ (hr)
Total CPT-11	60, *N* = 3	28.9 ± 15.8	2.4 ± 0.7	1047 ± 1210	1047 ± 1210	2.80 ± 1.59	136 ± 116	21.1 ± 11.7
	80, *N* = 6	29.2 ± 5.2	2.1 ± 0.7	1096 ± 834	1151 ± 880	3.39 ± 0.74	124 ± 106	33.3 ± 15.1
	100, *N* = 5	44.1 ± 7.7	4.0 ± 3.8	2237 ± 1090	2289 ± 1119	2.86 ± 0.75	58 ± 37	43.17 ± 4.8
	120, *N* = 2	47.9 ± 16.2	2.3 ± 0.9	1254 ± 553	1254 ± 553	3.95 ± 0.83	106 ± 47	54.4 ± 17.4
SN-38	60, *N* = 3	7.02 ± 5.64	13.1 ± 11.7	364 ± 222	1370 ± 1122	NA	NA	183.8 ± 172.3
	80, *N* = 6	7.98 ± 4.39	13.3 ± 18.3	343 ± 133	505 ± 165	NA	NA	57.5 ± 17.8
	100, *N* = 5	7.39 ± 1.68	12.2 ± 12.3	539 ± 368	840 ± 433	NA	NA	73.4 ± 18.3
	120, *N* = 2	7.26 ± 3.90	37.8 ± 17.2	353 ± 164	305	NA	NA	30.8
	Irinotecan^a^	26.3 ± 11.9	NA	229 ± 108	NA	NA	NA	10.4 ± 3.1

Mean ± STD; *C*_*max*_, peak concentration in plasma; *T*_*max*_, time to achieve peak plasma concentration; AUC_0→169.5_ and AUC_0→∞_, area under the plasma concentration-time curve from time zero to 169.5 h and infinity, respectively; V_*ss*_, volume of distribution at steady state; *t*_*1/2*_, plasma terminal elimination half-life; Cl, total clearance of drug from plasma; NA, not available

^a^Irinotecan 125 mg/m^2^, package inset of Campto^®^

The majority of subjects showed wild type alleles for UGT1A1*28 (TA6TA6: 88%) and UGT1A1*6 (GG: 69%). No subject harbored homozygous mutation in UGT1A1*28 or UGT1A1*6 allele. Two and five patients had heterozygous UGT1A1*28 and UGT1A1*6, respectively. Of which, one patient with heterozygous UGT1A1*28 and UGT1A1*6 experienced grade IV neutropenia and grade III diarrhea, and had the largest dose-normalized AUC of SN-38. Four out of the 5 subjects with heterozygous UGT1A1*6 possessed relatively higher dose-normalized AUC of SN-38 comparing to other subjects; of which 3 patients experienced grade III toxicities.

### Antitumor activity

One patient at dose level III, who suffered from DLT did not complete at least one post-treatment tumor assessment. Among the 15 efficacy evaluable patients, two (13.3%) had confirmed partial response (PR) and nine (60%) had stable disease (SD), leading to the overall disease control rate (DCR) of 73.3%. At the MTD of 80 mg/m^2^, 1 PR and 4 SD were observed among six patients. The tumor response rate and the disease control rate were 16.7 and 83.3%, respectively. PR was observed in one gastric cancer patient (at the 80 mg/m^2^ dose level) and one breast cancer patient (at the 100 mg/m^2^ dose level).

## Discussion

The current study evaluated the safety profile and preliminary efficacy of PEP02 in combination with 5-FU and LV, in patients with refractory advanced malignancy. Gastrointestinal toxicities and myelosuppression were the major DLTs, which were comparable to those of free irinotecan and PEP02 monotherapy [3, 9]. The MTD (80 mg/m^2^) of PEP02, in combination with infusion of 5-FU and LV on days 1 and 8 of every-3-week schedule is recommended for the future studies. In a previous study, the MTD of PEP02 monotherapy with a 3-week interval was 120 mg/m^2^ [3]. The favorable toxicity profiles of PEP02 made it a better agent to combine with other cytotoxic agents. 5-FU/LV in combination with irinotecan was the first line treatment of colorectal cancer, which explains our interest in the evaluation of PEP02 in combination with 5-FU/LV. The dose of weekly 5-FU in this study was fixed as 2000 mg/m^2^, which mimicked the AIO regimen commonly used in Europe and Asia [10, 11]. The percentage of grade III or above AEs or all treatment-related AEs in the MTD group was lower than that in the overall safety population. For hematologic laboratory parameters, nadir was observed between days 13 and 16 after PEP02 administration; however blood biochemistry was mostly unaffected. These tolerable and manageable hematological and non-hematological toxicities indicated that this combination therapy is feasible for further application.

PEP02 affected the PK characteristics of irinotecan. Compared to the data of 125 mg/m^2^ free-form irinotecan, 80 mg/m^2^ of PEP02 showed lower Cmax (8.0 ± 4.4 ng/mL vs. 26.3 ± 11.9 ng/mL), longer terminal t1/2 (57.5 ± 17.8 h vs. 10.4 ± 3.1 h) and higher AUC (343 ± 133 ng/mL*hr vs. 229 ± 108 ng/mL*hr) of SN-38 [12, 13]. These favorable PK parameters indicated that PEP02 could decrease the influx of SN-38 from the central compartment to the peripheral, leading to less treatment-related toxicities, even in combination with 5-FU/LV. The PK data showed the dose-dependent linear distribution of CPT-11 when study doses were increased from 60 to 120 mg/m^2^, but no statistically significant difference was observed in the mean values of pharmacokinetic parameters of CPT-11 and SN-38, including dose-normalized C_max_, AUC parameters, t_1/2_, CL, and V_ss_, possibly owning to narrow dose increments, small sample size and high inter-individual variability.

The *UGT1A1* gene encoded a varied spectrum of active enzymes that are responsible for drug metabolism, including UGT. The UGT1A1*28 allele is characterized by the presence of a 7th dinucleotide repeat in the TATA box of the promoter region, compared to the UGT1A1*1 allele with 6 repeats. This increased number of repeats results in the reduction in the expression of UGT, leading to decreased SN-38 detoxification and prolonged exposure time of SN-38 in the intestines. Thus, patients with homozygous or heterozygous UGT1A1*28 and treated with irinotecan commonly developed dose limiting neutropenia and late diarrhea [14]. Similar to UGT1A1*28 polymorphism, the UGT1A1*6 allele also can decrease the activity of the enzyme in the heterozygous or homozygous genotype. It has been reported that patients with both UGT1A1*28 and UGT1A1*6 heterozygosity were at high risk to develop irinotecan-related toxicities [15, 16]. In our study, owning to the small sample size, a clear correlation cannot be obtained between polymorphism of *UGT1A* family genes and pharmacokinetic parameters or toxicity of PEP02. However, one subject with heterozygous mutation in both UGT1A1*6 and UGT1A1*28 had the highest dose-normalized AUC of SN-38 and experienced grade IV neutropenia and grade III diarrhea. To draw any firm conclusions, a PK/PD study according to polymorphism of *UGT1A* family genes should be performed [17].

With the limitation of being a very small sample size study of 15 efficacy evaluable population, two subjects had confirmed PR and nine subjects had SD as their best-ever responses during this study period. The tumor response rate and disease control rate were 13 and 73%, respectively. In a Phase I trial, clinical efficacy cannot be defined accurately because of heterogeneous tumor types and different dose levels. Of the evaluable patients, PR was noted in a heavily treated breast cancer patient and a gastric cancer patient, and four out of five patients with pancreatic cancer had SD, implying that this combination regimen is worthy of further investigation. Indeed, PEP02 either alone or in combination with 5-FU/LV was investigated in a phase II PEP0208 study [18] and a phase III NAPOLI-1 study [19] in metastatic pancreatic cancer patients who progressed after gemcitabine-containing regimen. The NAPOLI-1 study formed the basis for the regulatory approvals of PEP02 (Irinotecan liposome injection) by the Taiwan FDA and US FDA in October 2015.

## Conclusions

This is the first trial to apply PEP02 in combination with 5-FU and LV in patients with solid tumors, and major treatment-related DLTs were myelosuppression and diarrhea. PEP02 had a lower C_max_, longer t_1/2_ and increased AUC_0→t_ of SN-38 compared to irinotecan; similar results were observed in another study on PEP02 infusion alone. The dose of 80 mg/m^2^ of PEP02 in combination with D1 and D8 infusion of 5-FU/LV with every-3-week schedule is recommended for future studies.

## Additional file

Additional file 1: Table S1. Tumor type, dose level, DLT, best response and single nucleotide polymorphisms of UGT1A1^*^28 and UGT1A1^*^6. (DOCX 18 kb)

## Abbreviations

5-FU: 5-fluorouracil
AEs: Adverse events
ANC: Absolute neutrophil count
AUC_0→∞_: AUC through infinite time
AUC_0→t_: Plasma concentration-time curve from zero to time t
CL: Clearance
C_max_: Peak plasma concentration
CPT-11: Irinotecan hydrochloride
CT: Computed tomography
DLT: Dose-limiting toxicity
ECOG: Eastern Cooperative Oncology Group
FDA: The Food and Drug Administration
LV: Leucovorin
MTD: Maximum tolerated dose
PG: Pharmacogenetics
PK: Pharmacokinetics
PR: Partial response
PS: Performance score
SD: Stable disease
SN-38G: SN-38 glucuronide
STD: Standard deviation
t_1/2_: Elimination half-life
T_max_: Time at which C_max_ occurred
UGT1A: UDP-glucuronosyl transferase 1A isoforms
V_*ss*_: Volume of distribution at steady state
