# Supplementary material for: A phase I dose-escalation study of PEP02 (irinotecan liposome injection) in combination with 5-fluorouracil and leucovorin in advanced solid tumors
Source: BMC Cancer. 2016 Nov 21;16:907. doi: 10.1186/s12885-016-2933-6 (PMC5117585; doi:10.1186/s12885-016-2933-6)
Supplement: Additional file 1: Table S1. — Tumor type, dose level, DLT, best response and single nucleotide polymorphisms of UGT1A1*28 and UGT1A1*6. (DOCX 18 kb) [file 12885_2016_2933_MOESM1_ESM.docx]

**Information supporting the conclusion:**

| **Tumor type** | **Dose level (mg/m^2^)** | **DLTs** | **DLT type** | **Best response** | ***UGT1A1*28*** | ***UGT1A1*6*** |
| --- | --- | --- | --- | --- | --- | --- |
| Breast ca. | 60 | No | - | PD | (TA)6/(TA)6 | G/G |
| SqCC | 60 | No | - | N/A | (TA)6/(TA)6 | G/G |
| Pancreatic Ca. | 60 | No | - | SD | (TA)6/(TA)6 | G/G |
| Gastric ca. | 80 | No | - | SD | (TA)6/(TA)6 | G/A |
| Pancreatic ca. | 80 | No | - | SD | (TA)6/(TA)6 | G/A |
| Nasopharyngeal ca. | 100 | No | - | SD | (TA)6/(TA)6 | G/G |
| Breast ca. | 100 | No | - | PR | (TA)6/(TA)6 | G/A |
| Gastric ca. | 100 | Yes | grade III infection with hypotension and grade III hemorrhage | PD | (TA)6/(TA)6 | G/G |
| Pancreatic ca. | 120 | Yes | grade III diarrhea and grade IV neutropenia | SD | (TA)6/(TA)6 | G/G |
| Breast ca. | 120 | Yes | grade III diarrhea | SD | (TA)6/(TA)6 | G/G |
| Cervical ca. | 100 | No |  | SD | (TA)6/(TA)6 | G/G |
| Breast ca. | 100 | Yes | grade III diarrhea and grade IV neutropenia | N/A | (TA)7/(TA)6 | G/A |
| Gastric ca. | 80 | No | - | PR | (TA)7/(TA)6 | G/G |
| Pancreatic ca. | 80 | No | - | SD | (TA)6/(TA)6 | G/A |
| Pancreatic ca. | 80 | No | - | PD | (TA)6/(TA)6 | G/G |
| Gastric ca. | 80 | No | - | SD | (TA)6/(TA)6 | G/G |

DLT, dosing limiting toxicity; Ca., carcinoma; SqCC, squamous cell carcinoma; PD, progressive disease; N/A, not available; SD, stable disease; PR, partial regression.
